# Public stated preferences and predicted uptake for genome-based colorectal cancer screening

**DOI:** 10.1186/1472-6947-14-18

**Published:** 2014-03-19

**Authors:** Catharina GM Groothuis-Oudshoorn, Jilles M Fermont, Janine A van Til, Maarten J IJzerman

**Affiliations:** 1 Department of Health Technology and Services Research, MIRA Institute for Biomedical Technology & Technical Medicine, University of Twente, P.O. box 217, 7500 AE, Enschede, The Netherlands; 2 Health Economics Research Centre, Nuffield Department of Population Health, University of Oxford, Oxford, United Kingdom

**Keywords:** Discrete choice experiment, Conjoint analysis, Nanopill, Colorectal cancer screening, Health technology assessment

## Abstract

**Background:**

Emerging developments in nanomedicine allow the development of genome-based technologies for non-invasive and individualised screening for diseases such as colorectal cancer. The main objective of this study was to measure user preferences for colorectal cancer screening using a nanopill.

**Methods:**

A discrete choice experiment was used to estimate the preferences for five competing diagnostic techniques including the nanopill and iFOBT. Alternative screening scenarios were described using five attributes namely: preparation involved, sensitivity, specificity, complication rate and testing frequency. Fourteen random and two fixed choice tasks, each consisting of three alternatives, were offered to 2225 individuals. Data were analysed using the McFadden conditional logit model.

**Results:**

Thirteen hundred and fifty-six respondents completed the questionnaire. The most important attributes (and preferred levels) were the screening technique (nanopill), sensitivity (100%) and preparation (no preparation). Stated screening uptake for the nanopill was 79%, compared to 76% for iFOBT. In the case of screening with the nanopill, the percentage of people preferring not to be screened would be reduced from 19.2% (iFOBT) to 16.7%.

**Conclusions:**

Although the expected benefits of nanotechnology based colorectal cancer screening are improved screening uptake, assuming more accurate test results and less preparation involved, the relative preference of the nanopill is only slightly higher than the iFOBT. Estimating user preferences during the development of diagnostic technologies could be used to identify relative performance, including perceived benefits and harms compared to competitors allowing for significant changes to be made throughout the process of development.

## Background

Approximately 436,000 people in Europe are newly diagnosed with colorectal cancer (CRC) annually [1]. Worldwide this figure reaches more than one million and these numbers are expected to increase with an ageing population [2]. In most European countries the 5-year survival rate is less than 60% [3]. If CRC is diagnosed at an early stage, the 5-year survival rate increases to almost 90% [4]. Screening is carried out, dependent on local guidelines, using immunochemical faecal occult blood test (iFOBT), colonoscopy, sigmoidoscopy, virtual colonoscopy or double contrast barium enema [5]. Population-based screening, using one of these methods, is recommended in most countries. The requirements for a test to be used as a screening instrument include high sensitivity, low cost and low burden to the participant.

Improved technology combined with increased knowledge and understanding of DNA sequencing has led to a better understanding of the aetiology of common diseases such as CRC and the potential use of biomarkers in disease detection. Several studies reporting the use of biomarkers in blood samples and gastro-intestinal fluid have been published [6-8]. For instance, the nanopill is a foresight of a digestible pill with diagnostic capabilities on molecular level [9]. The nanopill may be an alternative to current CRC screening modalities such as the iFOBT and sigmoidoscopy, with the promise of improved test performance and decreased burden of screening to the participant. The biggest difference between standard screening modalities and the nanopill is that the latter does not look for a tumour but rather it screens the participant’s gastro-intestinal fluid for hypermethylated DNA as a cancer marker. If the technology is being developed, it is suggested that the nanopill could be taken at home with minimal preparation.

Since the nanopill is still in development, the actual benefits in terms of increased test performance and health impact are unknown. In medical product development, decisions with regard to future development have to be made during the product development cycle. These decisions may benefit from early assessment of the potential of the product to compete in the healthcare market. Health Technology Assessment (HTA) is mostly carried out when a technology is fully developed and little to no adjustments can be made, possibly resulting in bad investments and product failure [10]. In contrast, an early assessment allows for timely decision-making so that significant product changes can still be made throughout the process of development [11].

In the case of the nanopill, it is important to understand the added value compared to its competitors and predictors for screening uptake in order to determine priorities in product development and targeting. Uptake rates for population-based CRC screening programs range between approximately 20% (Czech Republic) [12] and 70% (Finland) [13] for FOBT. Despite its favourable effect, the uptake for CRC screening remains generally poor, which has been variably accounted for by patient-related barriers [14-17], physician factors [18,19] and system failure [20,21]. As the population benefit and acceptance of screening programs largely depend on participation rates, the public perception of screening benefits and harms are important to estimate the potential of the technology in this intended target area. Current screening programs for CRC could be improved with regard to accuracy, burden, risks and required preparation [22-24].

The first objective of the study was to estimate public preferences for test characteristics (attributes) of screening technology and their relative importance in judging the overall attractiveness of a screening test. The second objective of the study was to estimate the predicted uptake of screening with the nanopill compared to its competitors while taking into account the attributes of the test.

## Methods

### Study population

The study was conducted among the general population in the Netherlands (NL) and the United Kingdom (UK). In contrast to the NL, a population-based CRC screening programme has already been implemented in the UK.

The sample was selected from an international Internet panel maintained by Survey Sampling International (SSI) in May 2011. SSI randomly recruited and invited 2225 respondents from the NL and the UK by email. Inclusion criteria were based on the advice for CRC screening given by the council of the European Union (EU) [25]. Both men and women aged between 50 and 74 years with absence of CRC were eligible. To prevent duplicates, SSI used respondent verification. For all questionnaires unique one-click links were created allowing respondents to directly access the survey. Respondents were able to save their progress at any time and complete the questionnaire within one month after invitation. According to the NHS Health Research Authority information, this type of study did not require approval from an ethics committee in the UK. The internet survey data was collected following ESOMAR codes and guidelines.

### Sample size

For this type of study it is recommended to have a minimum sample size of 300 [26]. We aimed for at least 800 respondents per country in total to increase the power of the study and to anticipate on an expected large non-response. In total 2225 respondents were invited to participate, 1100 (49%) in the UK and 1125 (51%) in the NL.

### Study measures

The questionnaire consisted of two parts. In the first part preferences of respondents with regard to different CRC screening modalities were elicited with a discrete choice experiment (DCE). In the second part socio demographics and other characteristics were asked: gender, age, marital status, employment status, educational level, family history of CRC, screening experience, current health status, and perceived individual risk of CRC. The expected time of questionnaire completion was approximately twenty minutes.

### DCE construction

The current study employs an approach called DCE or conjoint analysis to elicit the general public’s preferences for screening programs. The DCE is based on random utility theory and is consistent with Lancaster’s economic theory of value [27,28]. Random utility theory allows the researcher to elicit preferences for complex multidimensional goods or services, from which models of (relative) preferences for different attributes of a good or service can be estimated [29,30].

In this study, respondents were presented with multiple three-profile choice sets (triplets). Each choice set consisted of a random combination of attribute levels that spans the full range of actual to perfect performance of the different diagnostic techniques for CRC. An example of the questionnaire and the descriptions of the different screening tests can be found in the Additional file 1 and Additional file 2.

The design of the DCE was based on good research practices for conjoint analysis [31]. The choice for the attributes and levels for the DCE were based on previous preference studies on CRC screening [32-37]. Attributes of the diagnostic tests reported in the literature that were considered important in assessing test performance according to clinicians, researchers, policy- and decision-makers were included. The six attributes with associated levels included in this study were: preparation (no preparation, laxatives, enemas, diet plus laxatives), technique (stool, short tube, long tube with sedation, pill), sensitivity (70%, 80%, 90%, 100%), specificity (70%, 80%, 90%, 100%), complication rate (none, 1/10.000, 10/10.000, 100/10.000), and the required frequency of testing (annual, biennial, every 5 years, every 10 years). It was decided to replace the attribute “process” as used in many other studies by “technique”, as this would better indicate the technology. The process characteristics of each test were given in the study information sheet. Another important attribute found in the literature is discomfort. However, since attribute independence is important discomfort was not included as it was also captured by the attributes “process” and “preparation”. Except for frequency, all attributes are product related characteristics. Testing frequency is an important factor for feasibility and cost-effectiveness of screening. Levels for frequency were related to frequencies found in existing CRC screening programs within the EU. Costs were not included as an attribute because in the UK there are no out-of-pocket costs for the CRC population-based screening programme and if a population-based screening would be introduced in the Netherlands, like other current screening programs, public resources would be used to cover the financial cost.

Combining six attributes with four levels each resulted in 4096 (4^6^) different combinations that could be used as different hypothetical screening profiles. There are 11.4 ^.^10^9^ possible combinations of three-alternative choice sets. Sawtooth SSI Web System v.7.0 was used to generate 999 unique questionnaires, each with fourteen profile pairs randomly drawn from a fractional factorial design with a balanced overlap. Balanced overlap was used to add some degree of level overlap, without duplicating scenarios in choice tasks, to provide an opportunity for more discrimination for when people use non-compensatory rules and to improve the precision of estimates of interaction terms.

**Figure 1:**
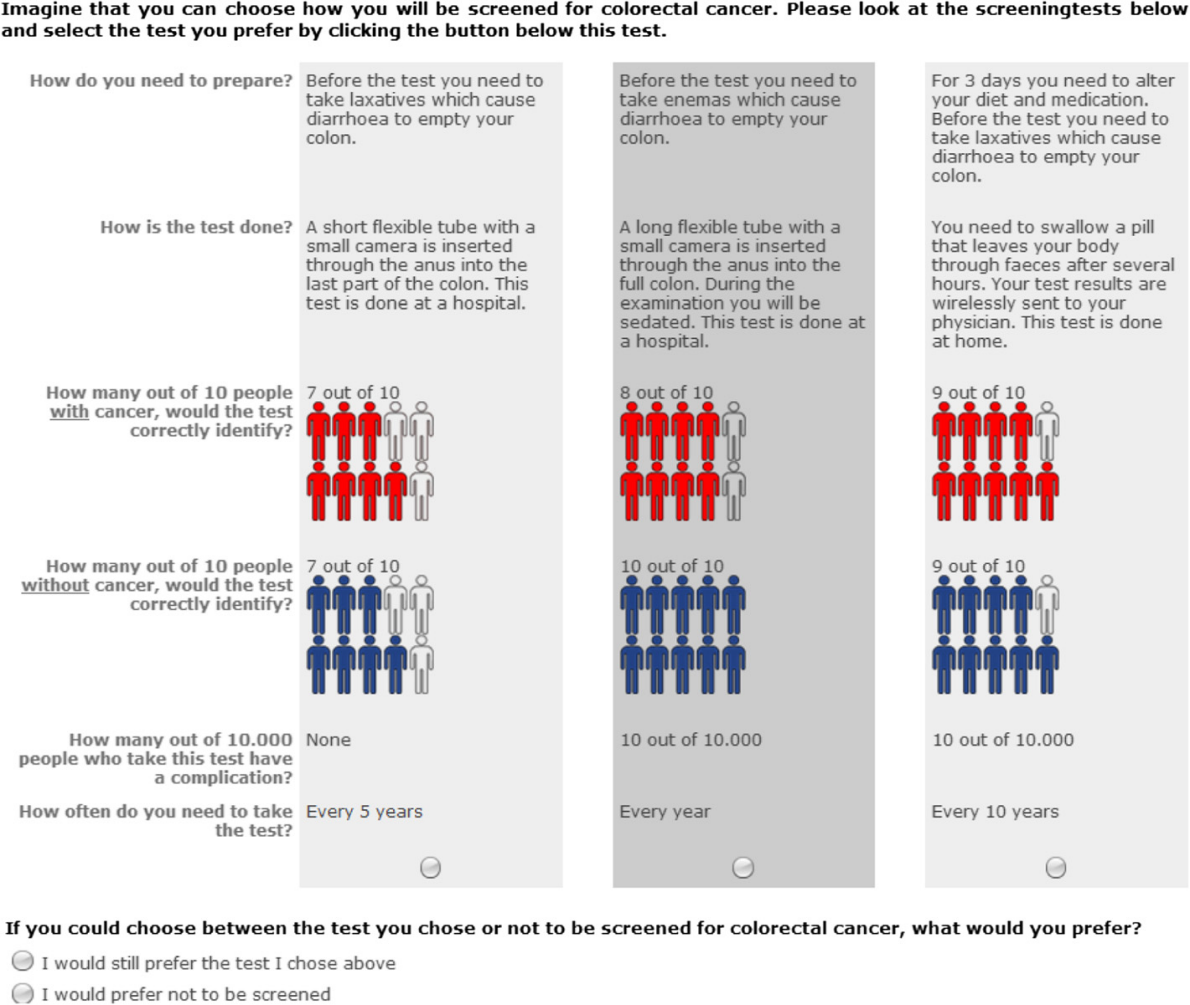
Choice set example.

Respondents were asked to imagine themselves eligible for CRC screening. In each question, respondents were asked to state their preferred scenario for CRC screening from a set of three hypothetical screening tests (Figure 1). After a decision was made, respondents were also offered not to participate in screening and were asked to choose between the previous chosen test and no screening. This type of dual-none response is called DR-2Max because it is a choice between two alternatives: the preferred and “none” [38]. The dual-none response question was included to prevent overestimation of screening uptake by assuming that all respondents would actually use the preferred test [39]. An example scenario and a choice set with response instructions were included to help respondents fill in the questionnaire.

### Pilot testing

Prior to distributing the questionnaire a pilot was conducted with thirty respondents. It was found that all respondents could evaluate the sixteen full-profile choice tasks in a reasonable amount of time but the phrasing of the attributes sensitivity, specificity and preparation was adjusted slightly to increase the understanding of the questions.

### Statistical analysis

Characteristics of the two samples, i.e. socio demographics, screening experience, perceived risk and family history of CRC, and health status were described using percentages for categorical variables and the mean (sd) for continuous variables. Differences between the two samples (NL and UK) were tested with Chi-square tests and t-tests.

Data of the DCE was analysed by stacking the dual responses for each respondent [38]. The response to the first choice was set up as a forced choice task between three scenarios and the second response was set up as a choice task among four alternatives including “none”. For respondents where the second choice was not “none” the first choice set was redundant and removed from the dataset. The stacked data was analysed using a conditional logit model.

We estimated the following model for the latent utility U for a CRC screening alternative:

$$U=V+\epsilon =\beta_{0}+\sum_{i=1}^{24}\beta_{i}x_{i}+\epsilon$$

V is an explainable, systematic term specified as a linear function of the CRC screening alternatives. x_i_ has a value of one if the associated level of a certain attribute is present in the particular screening alternative, -1 if the associated level is present and belongs to the reference level of the attribute and zero otherwise. β_i_ are the part-worth utility parameters for all levels of the attributes. ϵ is the random error, representing the individual variation in preferences. The constant term β_0_ is an “alternative specific constant”, indicating the relative weight on average placed by individuals on screening programmes compared to no screening. It is assumed that each individual chooses the screening alternative that maximises her/his utility (U) amongst the three alternatives in the choice set. Note that the utility (U) is not a cardinal utility on a scale between 0 and 1. In fact one can only interpret relative utility differences between scenarios.

Effects coding was used for the parameter estimation. Parameter coefficients, corresponding 95% confidence intervals and p-values are presented.

From the estimated utility scores, the expected uptake of a screening alternative *i* was predicted as the probability of accepting this screening alternative with the formula [40,41]:

$$P\left(\mathrm{Accepting}\;\mathrm{alternative}\;\mathrm{test}\;i\right)=\frac{1}{1+e^{-V_{i}}}.$$

The expected utility V_i_ of screening test i was calculated by adding the part-worth utilities for the different attributes corresponding with the different levels. This model assumes that an alternative with expected utility V equals zero has a probability of acceptance of 50%. Due to the effects coding, all parameters are estimated relative to the grand mean, which has an expected utility of zero. In other words: the mean indirect utility over all possible screening options is fixed at zero. In addition, this means that the expected uptake rates are estimated relative to the uptake rate of this grand mean.

Each competing screening alternative was represented by the attribute levels that approximates most closely with the actual values: iFOBT with no preparation, stool, 80% sensitivity, 90% specificity, no complication rate and biennial testing; sigmoidoscopy: preparation with enemas, short tube, 70% sensitivity, 90% specificity, 10 per 10.000 complication rate and screening every 5 years; colonoscopy: preparation with diet plus laxatives, long tube with sedation, 90% sensitivity, 90% specificity, a complication rate of 100 per 10.000 and a 10 year interval; nanopill: preparation with laxatives, pill, 100% sensitivity, 100% specificity, a complication rate of 1 per 10.000 and yearly testing*.* To estimate the minimal test requirements for the nanopill to be of additional benefit, trade-offs made by respondents were examined in a sensitivity analysis by varying the levels of the attributes. Finally, the probability of choosing between screening with test *i* or no screening was estimated with the multinomial logit model [42].

To study the effect of personal characteristics on the likelihood of choosing ‘no screening’, interactions between the alternative specific constant and the following categorical variables were included in the previous conditional logit model: country (NL vs UK), gender (female vs male), age (in years), marital status (married vs unmarried), employment status (full-time/part-time/self-employed vs homemaker/unemployed/retired), education (college/university vs less than college), family history (yes vs no/not known), screening experience (yes vs no), health status (excellent/(very) good vs fair/poor ), and risk perception (yes vs no). Effects of personal characteristics on preferences for attributes of the tests were not examined.

The fit of the models was assessed with McFadden’s pseudo R-squared and compared to each other with likelihood ratio chi-square statistics. Analyses were done in Stata v.11.0 with a significance level of p < 0.05.

## Results

### Study population

There were 1649 respondents in total from which 292 were excluded; 31 respondents due to the presence of CRC and 262 respondents did not fully complete the survey. This results in a response rate of 61% (1356 out of 2225). A response rate of 69% was achieved in the UK and 53% in the NL. The characteristics of the respondents are presented in Table 1. In total 11% (147 out of 1356) had a family history of CRC, 32% (438 out of 1356) had experience with CRC screening, and 23% (317 out of 1356) perceived themselves at risk for developing CRC.

**Table 1:** Self-reported characteristics of respondents (n = 1356)

**Characteristics**	**Total (%)**	**United Kingdom (%)**	**The Netherlands (%)**	
Response/invited	1649/2225 (74)	870/1100 (79)	779/1125 (69)	
Completed questionnaires	1356 (61)	763 (69)	593 (53)	p < 0.001
Gender				p = 0.842
Male	691 (51)	387 (51)	304 (51)	
Female	665 (49)	376 (49)	289 (49)	
Age (years)	60.5 (5.9)	60.7 (6.1)	60.2 (5.8)	p = 0.127
Marital status				p = 0.923
Married	896 (66)	505 (66)	391 (66)	
Not married	460 (34)	258 (34)	202 (34)	
Current employment status				p < 0.001
Full-time	248 (18)	137 (18)	111 (19)	
Part-time	167 (12)	82 (11)	85 (14)	
Self-employed	88 (7)	61 (8)	27 (5)	
Homemaker	150 (11)	51 (7)	99 (17)	
Unemployed	122 (9)	73 (10)	49 (8)	
Retired	581 (43)	359 (47)	222 (37)	
Education				p < 0.001
Public/primary school	96 (7)	63 (8)	33 (6)	
High school	605 (45)	260 (34)	345 (58)	
Trade/technical qualification	205 (15)	136 (18)	69 (12)	
College/university	450 (33)	304 (40)	146 (25)	
Family history CRC				p < 0.001
Yes	147 (11)	63 (8)	84 (14)	
No	1123 (83)	655 (86)	468 (79)	
Do not know	86 (6)	45 (6)	41 (7)	
Experience with screening				p < 0.001
Yes	438 (32)	325 (43)	113 (19)	
No	918 (68)	438 (57)	480 (81)	
Health status				p < 0.001
Excellent	105 (8)	49 (6)	56 (9)	
Very good	328 (24)	218 (29)	110 (19)	
Good	498 (37)	254 (33)	244 (41)	
Fair	333 (25)	181 (24)	152 (26)	
Poor	92 (7)	61 (8)	31 (5)	
Perceived risk developing CRC				p = 0.663
Yes	317 (23)	175 (23)	142 (24)	
No	1039 (77)	588 (77)	451 (76)	

In both countries the majority of respondents were retired, but the current employment status differed between the countries (χ2 = 47.7, 5df, p < 0.001). The mean age of the study sample was 60.5 (sd = 5.9). The respondent sample from the UK had a higher response rate (χ2 = 39.4, 1df, p < 0.001), were higher educated (χ2 = 78.6, 3df, p < 0.001) and had more screening experience (χ2 = 84.6, 1df, p < 0.001) compared to the Dutch sample. Respondents from the NL had a higher percentage of respondents with a positive family history of CRC (χ2 = 13.2, 2df, p = 0.001).

### DCE results

The estimated β-coefficients for the attribute levels are ordered as expected and statistically significant at the alpha <0.001 level, except for laxatives (p = 0.02), annual screening (p = 0.104) and screening every five years (p = 0.74), see Table 2. From the estimated part-worth utilities it can be concluded that respondents prefer a non-invasive test that is highly sensitive, requires no preparation, offered biennial, highly specific and has no complications. The least preferred screening combination is an invasive test that requires much preparation, has low sensitivity and specificity, a high screening interval and a high complication rate.

**Table 2:** Respondents preferences for colorectal cancer screening

**Attribute level**	**β − coefficient**	**95% CI**
Preparation		
No preparation	0.38 (0.02)^a^	0.34 to 0.42
Laxatives	−0.03 (0.01)^b^	−0.06 to -0.01
Enemas	−0.16 (0.01)^a^	−0.19 to -0.13
Diet plus laxatives^#^	−0.19 (0.02)^a^	−0.22 to -0.16
Technique		
Pill	0.48 (0.02)^a^	0.44 to 0.52
Stool	0.24 (0.02)^a^	0.20 to 0.28
Long tube with sedation^#^	−0.34 (0.02)^a^	−0.39 to -0.30
Short tube	−0.37 (0.02)^a^	−0.41 to -0.33
Sensitivity		
100%	0.36 (0.02)^a^	0.32 to 0.39
90%	0.08 (0.01)^a^	0.05 to 0.10
80%	−0.14 (0.01)^a^	−0.17 to -0.11
70%^#^	−0.30 (0.02)^a^	−0.33 to -0.26
Specificity		
100%	0.15 (0.01)^a^	0.12 to 0.17
90%	0.06 (0.01)^a^	0.03 to 0.09
80%	−0.04 (0.01)^a^	−0.07 to -0.02
70%^#^	−0.16 (0.02)^a^	−0.19 to -0.13
Complications		
None	0.14 (0.02)^a^	0.11 to 0.17
1/10.000	0.04 (0.01)^a^	0.01 to 0.06
10/10.000	−0.04 (0.01)^a^	−0.07 to 0.02
100/10.000^#^	−0.13 (0.02)^a^	−0.16 to -0.10
Frequency		
Biennial	0.17 (0.02)^a^	0.14 to 0.21
Annual^#^	0.03 (0.02)	−0.01 to 0.07
Every 5 years	0.01 (0.02)	−0.03 to 0.04
Every 10 years	−0.21 (0.02)^a^	−0.26 to -0.17
No screening	−0.29 (0.03)^a^	−0.36 to -0.23

^#^: reference level in effects coding; ^a^p-value < 0.005; ^b^p-value < 0.05; 65079 Observations, 1356 respondents. McFadden R^2^ = 11.0%.

The difference between the part-worth utilities for the levels indicates the utility that could be gained by changing (attaining) this level. As such, it can be concluded that the nanopill has the highest perceived utility. However, the utility to be gained from changing from iFOBT to nanopill screening is smaller than if an improvement from 90% to 100% sensitivity could be attained (0.28). When taking into account that iFOBT is currently seen as the screening test of choice, the highest improvement in utility by changing a single attribute of the test could be obtained by omitting the need for preparation (0.42).

The nanopill has the highest overall expected utility (1.31) compared to the other screening alternatives. The difference in utility between the nanopill and the iFOBT is 0.17, which is statistically significant (p = 0.006). The estimated utility for sigmoidoscopy (-0.51) and colonoscopy (-0.44) is smaller than for no screening (-0.29), meaning that no screening is preferred (on average) to sigmoidoscopy and colonoscopy.

**Figure 2:**
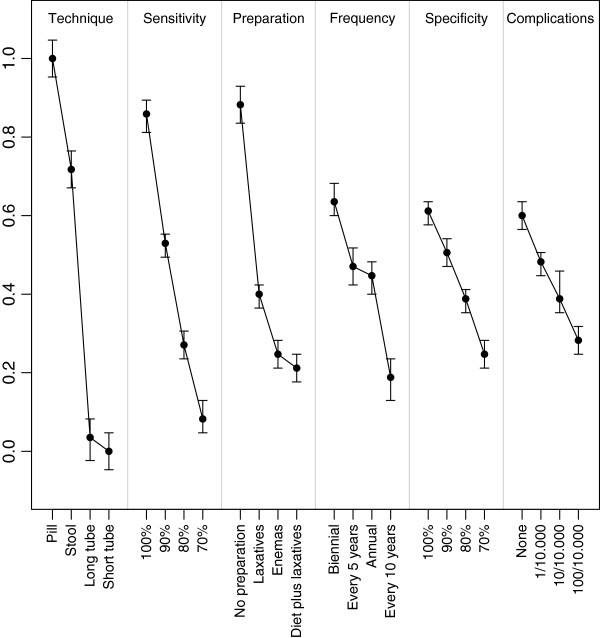
Relative importance of colorectal cancer screening attributes and attribute levels.

The absolute difference in the parameter estimates between attribute levels is the largest for technique, namely 0.85, which indicates that this is the most important attribute in the choice for screening tests. It is two to three times as large as the differences for specificity (0.31), complication rate (0.27) and frequency (0.38) and also larger than for preparation (0.57) and sensitivity (0.66). This implies that attributes related to the screening method (technique and preparation) appear to be more important than attributes related to the process of screening (complications and frequency). In Figure 2 the relative attribute importance and relative preference of levels of each attribute are given in order of the attribute importance such that 0 corresponds to the least desirable attribute level (long tube with sedation) and 1 with the most desirable attribute level (pill). Higher values indicate that the attribute level is preferred to levels with lower values. The wider the range, the more critical the attribute is in the decision making process of screening participation.

### Interaction model for no screening

A total of 93 (6.9%) of the respondents consequently chose not to be tested. In contrast, 908 (67.0%) of the respondents consistently selected screening. This indicates that for the majority of respondents, the decision to participate in CRC screening is made independent of the actual test, its performance or the perceived burden of testing involved. Family history (p = 0.024), screening experience (p < 0.001) and risk perception (p < 0.001) significantly interact with no screening (Table 3). In other words, the utility of screening is higher where there is a family history of CRC, screening experience and higher perceived risk of developing CRC themselves and thus respondents with these characteristics are more likely to choose in participate to screening. For the remaining 355 (26.2%) respondents, the preference for no screening over the preferred screening scenario varied between 6.3% and 93.8% of the choice tasks. In a subgroup analysis for this group, the attributes technique and preparation are by far the most important, and the effect of sensitivity is much smaller compared to the group as a whole. In 14.3% of all 21694 choice tasks no screening was preferred to one of the three screening alternatives. The estimated part-worth utilities estimates of the attribute levels are almost the same as in the model without the interaction terms. McFadden’s pseudo R for the interaction model equals 12.6% and the interaction model significantly improves the model fit (χ2 = 1038.5, p < 0.001).

**Table 3:** Predictors for screening participation

**Patient characteristic**	**β − coefficient (se) Interaction model**
No screening	0.05 (0.10)
Interaction terms with no screening	
Netherlands	−0.13 (0.07)
Male	−0.08 (0.07)
Perceived risk	−0.56 (0.10)^a^
Health status high/excellent	−0.02 (0.07)
Screening experience	−0.50 (0.09)^a^
Age	0.007 (0.007)
Family history	−0.29 (0.13)^a^
Education high	−0.06 (0.07)
Married	−0.06 (0.07)
Employed	−0.04 (0.08)
Likelihood ratio χ^2^	8414^a^
McFadden R^2^	12.6%

^a^p-value < 0.005.

### Expected uptake of different CRC screening alternatives

The expected average uptake of CRC screening was 57.3 % (CI 55.7% to 58.8%). The uptake for the annual screening with the nanopill would be 78.8% (CI 77.0% to 80.5%), which is 3 percentage points higher than for the iFOBT. Colonoscopy and sigmoidoscopy are the least favourable screening alternatives (Table 4).

**Table 4:** Predicted choice probabilities for the different screening alternatives with varying scenarios for the nanopill

**Screening alternative**	**Sensitivity**	**Specificity**	**Complication rate**	**Frequency**	**Predicted probability (95% CI)**
No screening	-	-	-	-	42.7% (41.2% to 44.3%)
Colonoscopy^a^	90%	90%	100/10000	10 years	39.2% (36.8% to 41.6%)
Sigmoidoscopy^b^	70%	90%	10/10000	5 years	37.5% (35.2% to 39.8%)
iFOBT^c^	80%	90%	None	2 years	75.8% (73.9% to 77.7%)
1: Nanopill^d^	100%	100%	1/10000	Annual	78.8% (77.0% to 80.5%)
2: Nanopill^d^	100%	100%	1/10000	2 years	81.0% (79.4% to 82.6%)
3: Nanopill^d^	100%	90%	1/10000	Annual	77.3% (75.4% to 79.2%)
4: Nanopill^d^	90%	100%	1/10000	Annual	73.8% (71.7% to 75.7%)
5: Nanopill^d^	95%	95%	1/10000	Annual	75.6% (73.7% to 77.4%)
6: Nanopill^d^	90%	95%	1/10000	2 years	75.6% (73.7% to 77.4%)

^a^Diet plus laxatives, long tube with sedation; ^b^Enemas, short tube; ^c^No preparation, stool; ^d^Laxatives, pill.

The test characteristics of the nanopill were hypothetical. Changing the frequency of taking the nanopill from annually to biennial would increase the expected uptake by 2.2 percentage points. The nanopill should have at least 90% sensitivity, 95% specificity and be used at a frequency of every two years to be equally attractive as biennial iFOBT testing. Assuming that CRC screening uses iFOBT, 19.2% of the respondents would choose not be screened. In case of screening with the nanopill, 16.7% of the respondents would prefer not to be screened.

## Discussion

In this study public preferences for the screening of CRC were elicited to understand the potential of new biomarker based screening approaches. The results of the study indicate that the attribute “technique” was most important followed by the “sensitivity” of the test. When assuming that the nanopill will outperform the iFOBT on sensitivity and specificity, the predicted uptake to screening with the nanopill is three percentage points higher than with the iFOBT.

Previous studies have found that the sensitivity of a test is the most important characteristic of a screening test in CRC [35,43]. The finding that sensitivity was not the most important criterion in this study might be explained by the framing of the levels i.e. the use of a “perfect” sensitivity level of 100% and/or the smaller contrast between the different levels for sensitivity (70% to 100% instead of 40% to 90% in other studies). Attribute importance is a function of the range of attribute levels and wider ranges almost certainly will result in higher relative importances [30,44]. Moreover, it is unclear whether the respondents actually understood the actual numerical rates that were presented instead of thinking of them as categories like ‘very low’, ‘low’, ‘moderate’ and ‘high’. Alternatively, the graphical presentation of sensitivity levels might have influenced interpretation [43]. Conversely, the high importance given to the technique might be related to the extensive qualitative information given about different screening techniques prior to the DCE questions than the other attributes. In addition, the attention of the nanopill as a novel technique for diagnosis might have resulted in higher utility of this level.

Previous studies using stated preference techniques such as DCE in early stage consumer research and new product development have resulted in a better understanding of consumer needs [45], quality improvement of products and services [46], reduced time to market, prevented wasting resources on producing and evaluating inappropriate prototypes [47], estimated preferences for services or technologies that are not yet available and optimized screening uptake [43].

The results of this study give insight in the trade-offs made by the public in valuing screening techniques and the effect of the screening technique on predicted uptake. The results of this study may support development decisions for the nanopill.

The results of this study confirmed to the developers of the nanopill that it is important to focus on sensitivity and preparation, as these are the most important attributes of screening, in order to meet the priorities of its future users and thereby increase the product success. However, the room for improvement is limited, it seems that the public is not as averse to the handling of stool samples as the developers believed, indicated by the small actual difference in utility between stool sample and no preparation.

Also, the currently proposed screening technique of the iFOBT performs reasonably well with regard to sensitivity while the costs are rather low. Although the costs of the pill are currently unknown, it is unlikely that such a technologically advanced method will become cheaper than the low tech iFOBT screening. The nanopill outperformed other diagnostic tests in part due to its promise of higher sensitivity. However, in the early stage of development of the device, it is unknown whether this promise can be realised. Moreover, sensitivity over time is more important for screening than single test sensitivity [24,48] and the sensitivity of iFOBT could be improved if this relatively cheap test is taken more frequent [49]. The results of this study indicate that frequency of testing is a less important decision characteristic for the population.

The results of this study have to be interpreted with caution. First, the issue of respondent consistency should be considered. In this study, two fixed choice tasks were included to test for internal respondent consistency (test–retest). It was decided not to exclude respondents who failed this test because these seemingly ‘irrational’ responses might actually be ‘rational’. Deleting them may induce sample selection bias and lead to a reduction in model efficiency [50]. To examine the effects of respondent’s consistency, a second analysis was performed including only respondents who passed the consistency test. The relative order of the three most important attributes (technique, sensitivity and preparation) did not change.

A second limitation of our study is the interpretation of stated preference techniques and its relationship with actual uptake of screening. The predicted uptake to screening in the study is high compared to actual uptake rates in CRC. The highest actual screening uptake documented was 71% (Finland) [13], which is lower than the predicted uptake to screening for iFOBt in this study. A comparable predicted uptake rate of 72% was found for biennial iFOBT screening in the Netherlands by van Dam [34]. The results of this study indicate that the decision to participate in screening is, for a large part, independent of the test characteristics itself. It may be more likely that this decision depends on the characteristics and circumstances of each individual.

Finally, it is known that the outcomes from a DCE in terms of part-worth utility and relative importance estimates depend on the choice of the attribute levels [44]. It is only when the attribute levels are a reflection of the true range of alternatives that the outcomes are reliable estimates of actual preferences. However, in the specific case of the nanopill, the actual performance of the novel technology is yet unknown and has to be determined [43]. The same holds true for the choice of comparators. We selected four comparators based on their frequency of use in the diagnosis of CRC. Other comparators such as virtual colonoscopy and video capsule endoscopy were not included in the study because they are not offered as standard screening tools within the EU.

## Conclusions

Despite the expected benefits of a nanopill based screening programme in terms of improved screening uptake, earlier diagnosis and more accurate test results, the preference for the nanopill compared to its competitors is only slightly higher. Although these findings agree with previous studies and suggest that the nanopill would be accepted by the public, developers should take into account the public preference for a high quality and low burden screening test. Estimates from user preferences during the development of diagnostic technologies could be used to identify relative performance, including perceived benefits and harms compared to competitors, allowing for changes to be made throughout the process of development.

## Abbreviations

CRC: Colorectal cancer; DCE: Discrete choice experiment; EU: European Union; HTA: Health Technology Assessment; iFOBT: immunochemical Fecal Occult Blood Test; NL: the Netherlands; UK: the United Kingdom; SD: Standard deviation; SE: Standard error.

## Competing interests

The authors declare that they have no competing interests.

## Authors’ contributions

CGO participated in the design of the study, performed the statistical analysis and drafted the manuscript, JMF designed the study, participated in the statistical analysis and drafted the manuscript, JT participated in the design of the study and helped to draft the manuscript, MIJ initiated the study, participated in the design of the study and helped to draft the manuscript. All authors read and approved the final manuscript.

## Pre-publication history

The pre-publication history for this paper can be accessed here:

http://www.biomedcentral.com/1472-6947/14/18/prepub

## Supplementary Material

Additional file 1 This file contains the descriptions of the different screening tests as displayed to the respondents. Click here for file

Additional file 2 **This file contains a sample of the questionnaire that was sent to the respondents.** Since we used a randomised design in Sawtooth for the conjoint questions, each respondent got different but the same number of choicesets to compare. Click here for file
