# Supplementary material for: Public stated preferences and predicted uptake for genome-based colorectal cancer screening
Source: BMC Med Inform Decis Mak. 2014 Mar 19;14:18. doi: 10.1186/1472-6947-14-18 (PMC4000055; doi:10.1186/1472-6947-14-18)
Supplement: Additional file 1 — This file contains the descriptions of the different screening tests as displayed to the respondents. [file 1472-6947-14-18-S1.pdf]

Appendix 1: Descriptions of the different screening tests

Nanopill

The nanopill is a non-invasive test that can be taken at home. Before the test the patient needs to take laxatives which cause diarrhoea to empty the colon. The patient swallows a digestible pill that leaves the body through faeces after several hours. With electrical currents it detects hypermethylated DNA that is used as a cancer marker. Test results will be wirelessly sent to the physician.

iFOBT

Immunochemical-based Faecal Occult Blood Test (iFOBT) can be done at home. iFOBTs detect intact human haemoglobin. It is a non-invasive test that does not require bowel preparation or dietetic restrictions. For two days individuals need to collect a sample from two separate stools with an applicator stick that is included in the test package. Samples need to be sent to the physician or laboratory by mail for analysis.

Sigmoidoscopy

Sigmoidoscopy is done at a hospital. It is invasive and requires the patient to take an enema which cause diarrhea to empty the colon. Sigmoidoscopy is an endoscopic examination; a short flexible tube with a small camera is inserted through the anus into the last part of the colon, until the splenic flexure.

Colonoscopy

Colonoscopy is done at a hospital. It is invasive and it requires the patient to take laxatives which cause diarrhea to empty the colon. For three days diet and medication need to be altered. Colonoscopy is an endoscopic examination; a long flexible tube with a small camera is inserted through the anus into the full colon. During the examination the patient will be sedated.
