# Supplementary material for: Public stated preferences and predicted uptake for genome-based colorectal cancer screening
Source: BMC Med Inform Decis Mak. 2014 Mar 19;14:18. doi: 10.1186/1472-6947-14-18 (PMC4000055; doi:10.1186/1472-6947-14-18)
Supplement: Additional file 2 — This file contains a sample of the questionnaire that was sent to the respondents. Since we used a randomised design in Sawtooth for the conjoint questions, each respondent got different but the same number of choicesets to compare. [file 1472-6947-14-18-S2.pdf]

Start

Username:

Password:

Introduction

UNIVERSITY OF TWENTE.

The following questionnaire is part of a research project that focuses on test preferences concerning colorectal (bowel) cancer screening. It will take about 20 minutes to complete the questionnaire. You will contribute to scientific knowledge, therefore your participation is valuable.

There will be 16 questions with different testscenarios. Each question shows three testscenarios from which you need to choose the test you prefer most. After you made a decision you can also choose not to be screened. Some graphical aids are used for clarification. Finally you will be asked a few personal questions.

Your participation is voluntarily. Your answers are entirely confidential and anonymous. With the presentation of the results only group results will be displayed, therefore individual data cannot be recognized.

YesNo

The next question is important for your participation.

Do you have colorectal (bowel) cancer?

YesNo=1 Yes

YesNo=2 No

0% 100%

Example

The following is an example of a testscenario.

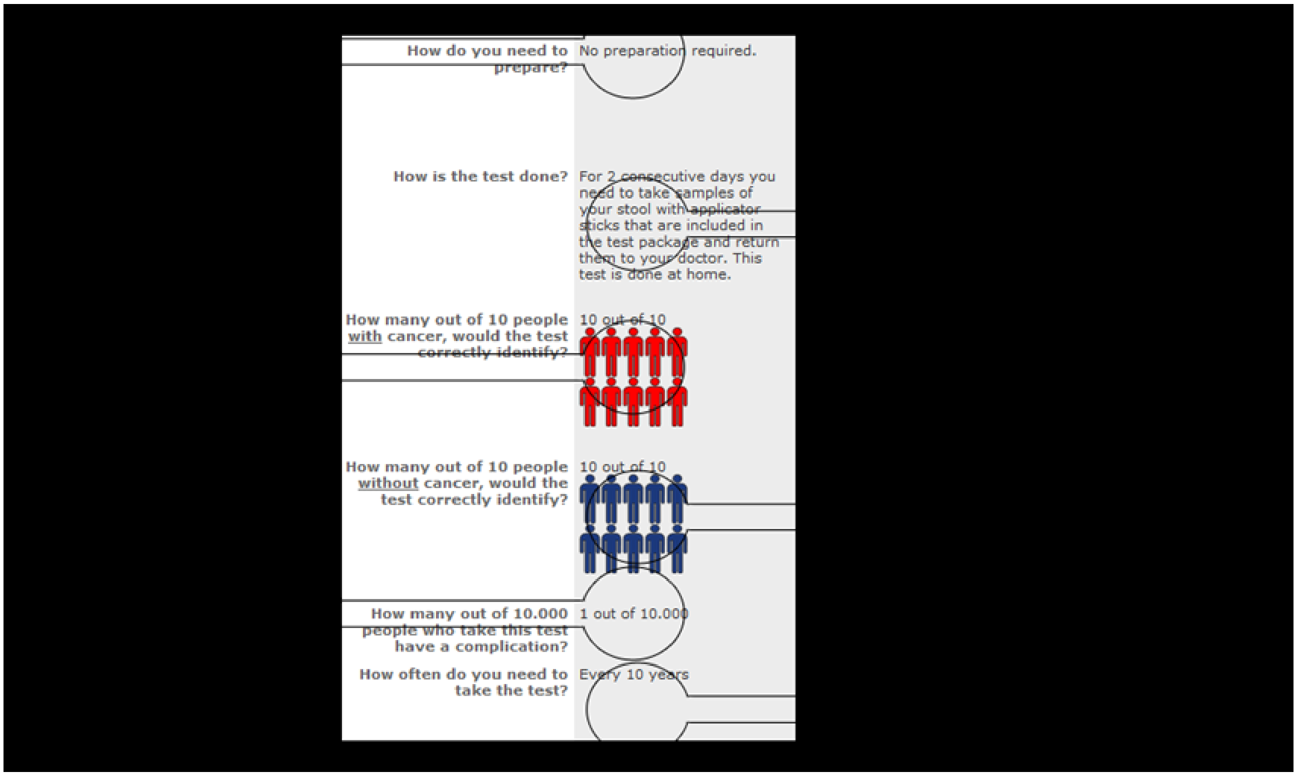

How do you need to prepare?	No preparation required.
How is the test done?	For 2 consecutive days you need to take samples of your stool with applicator sticks that are included in the test package and return them to your doctor. This test is done at home.
How many out of 10 people with cancer, would the test correctly identify?	10 out of 10
How many out of 10 people without cancer, would the test correctly identify?	10 out of 10
How many out of 10.000 people who take this test have a complication?	1 out of 10.000
How often do you need to take the test?	Every 10 years

0% 100%

Example1

The following is an <u>example</u> of a question. Please note that you have to make two decisions.

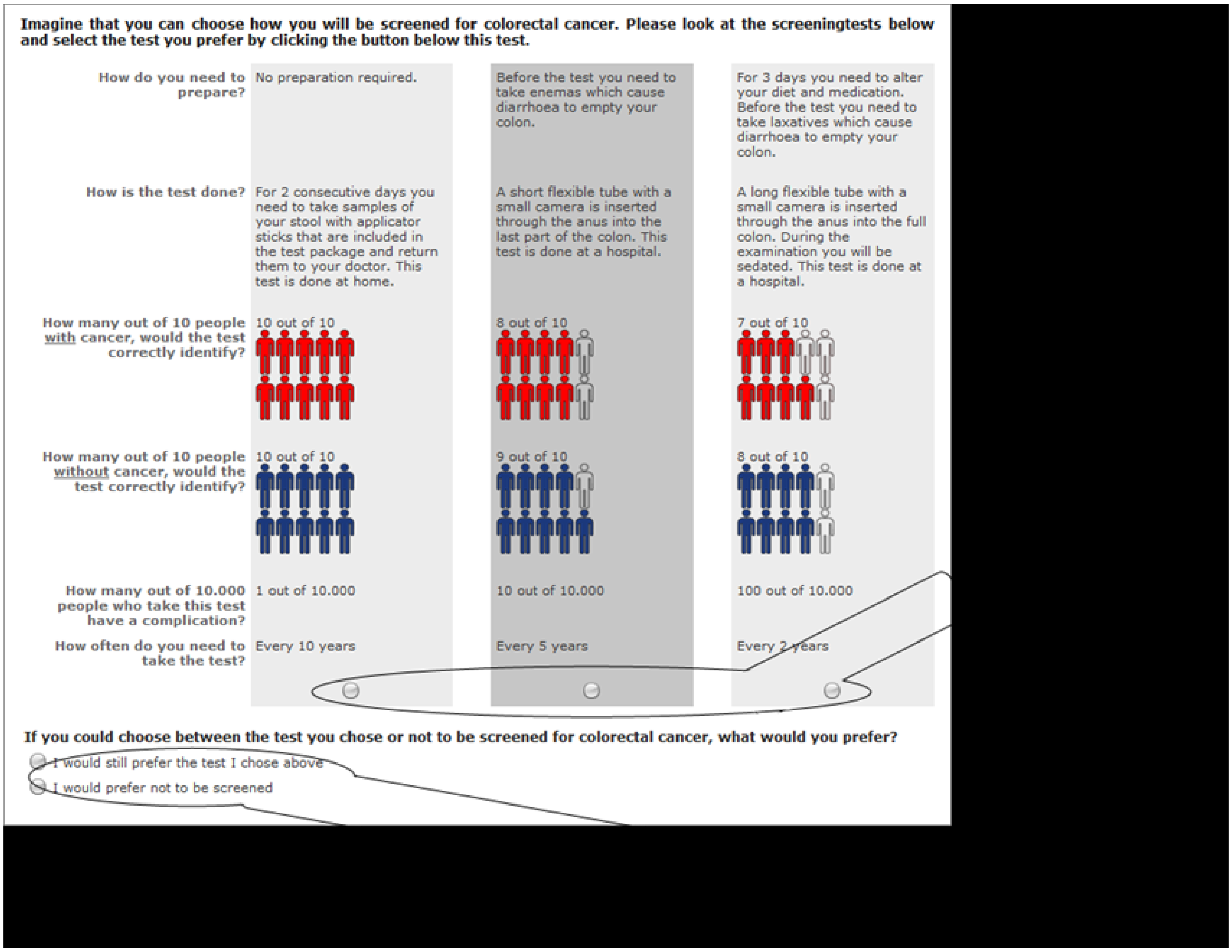

Imagine that you can choose how you will be screened for colorectal cancer. Please look at the screeningtests below and select the test you prefer by clicking the button below this test.

**How do you need to prepare?**	No preparation required.	Before the test you need to take enemas which cause diarrhoea to empty your colon.	For 3 days you need to alter your diet and medication. Before the test you need to take laxatives which cause diarrhoea to empty your colon.
**How is the test done?**	For 2 consecutive days you need to take samples of your stool with applicator sticks that are included in the test package and return them to your doctor. This test is done at home.	A short flexible tube with a small camera is inserted through the anus into the last part of the colon. This test is done at a hospital.	A long flexible tube with a small camera is inserted through the anus into the full colon. During the examination you will be sedated. This test is done at a hospital.
**How many out of 10 people <u>with</u> cancer, would the test correctly identify?**	10 out of 10	8 out of 10	7 out of 10
**How many out of 10 people <u>without</u> cancer, would the test correctly identify?**	10 out of 10	9 out of 10	8 out of 10
**How many out of 10.000 people who take this test have a complication?**	1 out of 10.000	10 out of 10.000	100 out of 10.000
**How often do you need to take the test?**	Every 10 years	Every 5 years	Every 2 years

If you could choose between the test you chose or not to be screened for colorectal cancer, what would you prefer?

- I would still prefer the test I chose above
- I would prefer not to be screened

The questionnaire starts hereafter!

0% 100%

CBC_Random1

Imagine that you can choose how you will be screened for colorectal cancer. Please look at the screeningtests below and select the test you prefer by clicking the button below this test.

How do you need to prepare?	Before the test you need to take laxatives which cause diarrhoea to empty your colon.	For 3 days you need to alter your diet and medication. Before the test you need to take laxatives which cause diarrhoea to empty your colon.	No preparation required.
How is the test done?	For 2 consecutive days you need to take samples of your stool with applicator sticks that are included in the test package and return them to your doctor. This test is done at home.	A short flexible tube with a small camera is inserted through the anus into the last part of the colon. This test is done at a hospital.	A long flexible tube with a small camera is inserted through the anus into the full colon. During the examination you will be sedated. This test is done at a hospital.
How many out of 10 people <u>with</u> cancer, would the test correctly identify?	10 out of 10 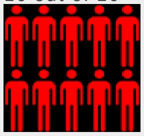	8 out of 10 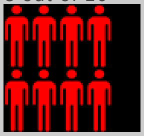	9 out of 10 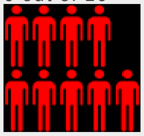
How many out of 10 people <u>without</u> cancer, would the test correctly identify?	7 out of 10 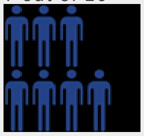	9 out of 10 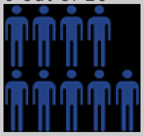	10 out of 10 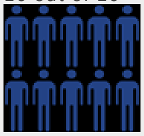
How many out of 10.000 people who take this test have a complication?	1 out of 10.000	None	100 out of 10.000
How often do you need to take the test?	Every 10 years	Every year	Every 2 years
	CBC_Random1=1	CBC_Random1=2	CBC_Random1=3

If you could choose between the test you chose or not to be screened for colorectal cancer, what would you prefer?

CBC_Random1_none=1 I would still prefer the test I chose above

CBC_Random1_none=2 I would prefer not to be screened

0% 100%

CBC_Random2

Imagine that you can choose how you will be screened for colorectal cancer. Please look at the screeningtests below and select the test you prefer by clicking the button below this test.

How do you need to prepare?	No preparation required.	Before the test you need to take enemas which cause diarrhoea to empty your colon.	Before the test you need to take laxatives which cause diarrhoea to empty your colon.
How is the test done?	You need to swallow a pill that leaves your body through faeces after several hours. Your test results are wirelessly sent to your physician. This test is done at home.	You need to swallow a pill that leaves your body through faeces after several hours. Your test results are wirelessly sent to your physician. This test is done at home.	A long flexible tube with a small camera is inserted through the anus into the full colon. During the examination you will be sedated. This test is done at a hospital.
How many out of 10 people <u>with</u> cancer, would the test correctly identify?	8 out of 10 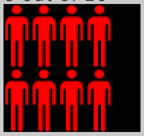	7 out of 10 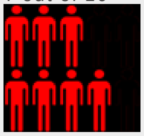	9 out of 10 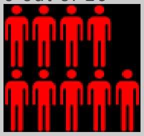
How many out of 10 people <u>without</u> cancer, would the test correctly identify?	8 out of 10 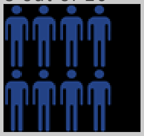	9 out of 10 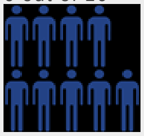	8 out of 10 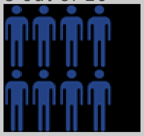
How many out of 10.000 people who take this test have a complication?	1 out of 10.000	10 out of 10.000	None
How often do you need to take the test?	Every year	Every 5 years	Every 10 years
	CBC_Random2=1 ○	CBC_Random2=2 ○	CBC_Random2=3 ○

If you could choose between the test you chose or not to be screened for colorectal cancer, what would you prefer?

CBC_Random2_none=1 ○ I would still prefer the test I chose above

CBC_Random2_none=2 ○ I would prefer not to be screened

0% 100%

CBC_Random3

Imagine that you can choose how you will be screened for colorectal cancer. Please look at the screeningtests below and select the test you prefer by clicking the button below this test.

How do you need to prepare?	Before the test you need to take laxatives which cause diarrhoea to empty your colon.	For 3 days you need to alter your diet and medication. Before the test you need to take laxatives which cause diarrhoea to empty your colon.	Before the test you need to take enemas which cause diarrhoea to empty your colon.
How is the test done?	For 2 consecutive days you need to take samples of your stool with applicator sticks that are included in the test package and return them to your doctor. This test is done at home.	A long flexible tube with a small camera is inserted through the anus into the full colon. During the examination you will be sedated. This test is done at a hospital.	A short flexible tube with a small camera is inserted through the anus into the last part of the colon. This test is done at a hospital.
How many out of 10 people with cancer, would the test correctly identify?	7 out of 10 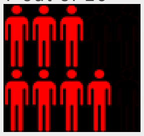	7 out of 10 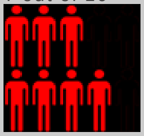	10 out of 10 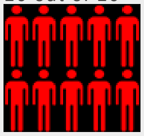
How many out of 10 people without cancer, would the test correctly identify?	10 out of 10 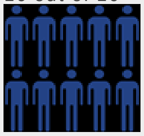	7 out of 10 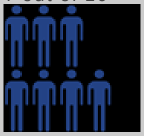	10 out of 10 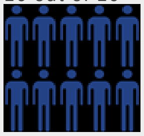
How many out of 10.000 people who take this test have a complication?	1 out of 10.000	100 out of 10.000	10 out of 10.000
How often do you need to take the test?	Every year	Every 5 years	Every 2 years
	CBC_Random3=1	CBC_Random3=2	CBC_Random3=3

If you could choose between the test you chose or not to be screened for colorectal cancer, what would you prefer?

CBC_Random3_none=1 I would still prefer the test I chose above

CBC_Random3_none=2 I would prefer not to be screened

0% 100%

CBC_Random4

Imagine that you can choose how you will be screened for colorectal cancer. Please look at the screeningtests below and select the test you prefer by clicking the button below this test.

How do you need to prepare?	Before the test you need to take enemas which cause diarrhoea to empty your colon.	No preparation required.	For 3 days you need to alter your diet and medication. Before the test you need to take laxatives which cause diarrhoea to empty your colon.
How is the test done?	A short flexible tube with a small camera is inserted through the anus into the last part of the colon. This test is done at a hospital.	You need to swallow a pill that leaves your body through faeces after several hours. Your test results are wirelessly sent to your physician. This test is done at home.	You need to swallow a pill that leaves your body through faeces after several hours. Your test results are wirelessly sent to your physician. This test is done at home.
How many out of 10 people <u>with</u> cancer, would the test correctly identify?	8 out of 10 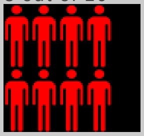	9 out of 10 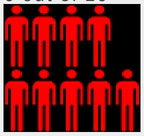	10 out of 10 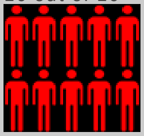
How many out of 10 people <u>without</u> cancer, would the test correctly identify?	7 out of 10 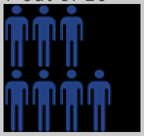	7 out of 10 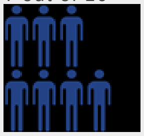	8 out of 10 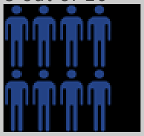
How many out of 10.000 people who take this test have a complication?	100 out of 10.000	10 out of 10.000	None
How often do you need to take the test?	Every 10 years	Every 5 years	Every 2 years
	CBC_Random4=1	CBC_Random4=2	CBC_Random4=3

If you could choose between the test you chose or not to be screened for colorectal cancer, what would you prefer?

CBC_Random4_none=1 I would still prefer the test I chose above

CBC_Random4_none=2 I would prefer not to be screened

0% 100%

CBC_Random5

Imagine that you can choose how you will be screened for colorectal cancer. Please look at the screeningtests below and select the test you prefer by clicking the button below this test.

How do you need to prepare?	Before the test you need to take enemas which cause diarrhoea to empty your colon.	Before the test you need to take laxatives which cause diarrhoea to empty your colon.	No preparation required.
How is the test done?	For 2 consecutive days you need to take samples of your stool with applicator sticks that are included in the test package and return them to your doctor. This test is done at home.	For 2 consecutive days you need to take samples of your stool with applicator sticks that are included in the test package and return them to your doctor. This test is done at home.	A short flexible tube with a small camera is inserted through the anus into the last part of the colon. This test is done at a hospital.
How many out of 10 people <u>with</u> cancer, would the test correctly identify?	9 out of 10 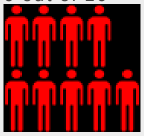	10 out of 10 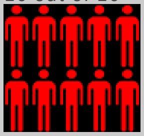	7 out of 10 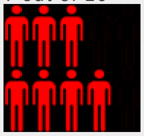
How many out of 10 people <u>without</u> cancer, would the test correctly identify?	9 out of 10 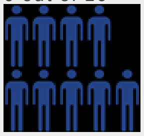	9 out of 10 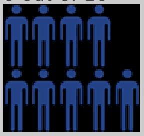	10 out of 10 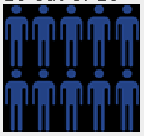
How many out of 10.000 people who take this test have a complication?	1 out of 10.000	100 out of 10.000	10 out of 10.000
How often do you need to take the test?	Every year	Every 5 years	Every 10 years
	CBC_Random5=1	CBC_Random5=2	CBC_Random5=3

If you could choose between the test you chose or not to be screened for colorectal cancer, what would you prefer?

CBC_Random5_none=1 I would still prefer the test I chose above

CBC_Random5_none=2 I would prefer not to be screened

0% 100%

CBC_Random6

Imagine that you can choose how you will be screened for colorectal cancer. Please look at the screeningtests below and select the test you prefer by clicking the button below this test.

How do you need to prepare?	No preparation required.	Before the test you need to take enemas which cause diarrhoea to empty your colon.	For 3 days you need to alter your diet and medication. Before the test you need to take laxatives which cause diarrhoea to empty your colon.
How is the test done?	A short flexible tube with a small camera is inserted through the anus into the last part of the colon. This test is done at a hospital.	You need to swallow a pill that leaves your body through faeces after several hours. Your test results are wirelessly sent to your physician. This test is done at home.	A long flexible tube with a small camera is inserted through the anus into the full colon. During the examination you will be sedated. This test is done at a hospital.
How many out of 10 people with cancer, would the test correctly identify?	7 out of 10 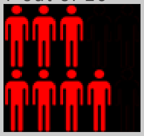	8 out of 10 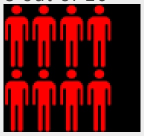	10 out of 10 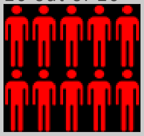
How many out of 10 people without cancer, would the test correctly identify?	8 out of 10 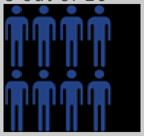	10 out of 10 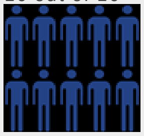	8 out of 10 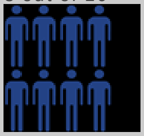
How many out of 10.000 people who take this test have a complication?	10 out of 10.000	1 out of 10.000	None
How often do you need to take the test?	Every year	Every 2 years	Every 2 years
	CBC_Random6=1	CBC_Random6=2	CBC_Random6=3

If you could choose between the test you chose or not to be screened for colorectal cancer, what would you prefer?

CBC_Random6_none=1 I would still prefer the test I chose above

CBC_Random6_none=2 I would prefer not to be screened

0% 100%

CBC_Fixed1

Imagine that you can choose how you will be screened for colorectal cancer. Please look at the screeningtests below and select the test you prefer by clicking the button below this test.

How do you need to prepare?	No preparation required.	Before the test you need to take enemas which cause diarrhoea to empty your colon.	For 3 days you need to alter your diet and medication. Before the test you need to take laxatives which cause diarrhoea to empty your colon.
How is the test done?	For 2 consecutive days you need to take samples of your stool with applicator sticks that are included in the test package and return them to your doctor. This test is done at home.	A short flexible tube with a small camera is inserted through the anus into the last part of the colon. This test is done at a hospital.	A long flexible tube with a small camera is inserted through the anus into the full colon. During the examination you will be sedated. This test is done at a hospital.
How many out of 10 people with cancer, would the test correctly identify?	10 out of 10 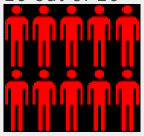	8 out of 10 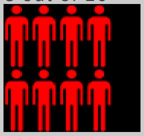	7 out of 10 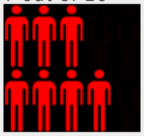
How many out of 10 people without cancer, would the test correctly identify?	10 out of 10 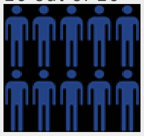	9 out of 10 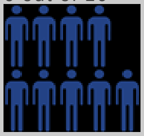	8 out of 10 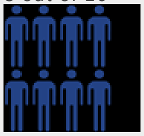
How many out of 10.000 people who take this test have a complication?	1 out of 10.000	10 out of 10.000	100 out of 10.000
How often do you need to take the test?	Every 10 years	Every 5 years	Every 2 years
	CBC_Fixed1=1	CBC_Fixed1=2	CBC_Fixed1=3

If you could choose between the test you chose or not to be screened for colorectal cancer, what would you prefer?

CBC_Fixed1_none=1 I would still prefer the test I chose above

CBC_Fixed1_none=2 I would prefer not to be screened

0% 100%

CBC_Random7

Imagine that you can choose how you will be screened for colorectal cancer. Please look at the screeningtests below and select the test you prefer by clicking the button below this test.

How do you need to prepare?	Before the test you need to take laxatives which cause diarrhoea to empty your colon.	For 3 days you need to alter your diet and medication. Before the test you need to take laxatives which cause diarrhoea to empty your colon.	For 3 days you need to alter your diet and medication. Before the test you need to take laxatives which cause diarrhoea to empty your colon.
How is the test done?	You need to swallow a pill that leaves your body through faeces after several hours. Your test results are wirelessly sent to your physician. This test is done at home.	For 2 consecutive days you need to take samples of your stool with applicator sticks that are included in the test package and return them to your doctor. This test is done at home.	A long flexible tube with a small camera is inserted through the anus into the full colon. During the examination you will be sedated. This test is done at a hospital.
How many out of 10 people with cancer, would the test correctly identify?	7 out of 10 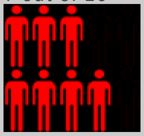	9 out of 10 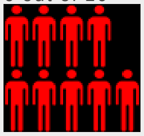	8 out of 10 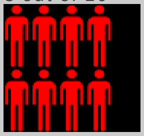
How many out of 10 people without cancer, would the test correctly identify?	10 out of 10 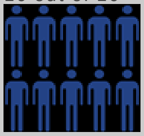	9 out of 10 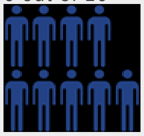	7 out of 10 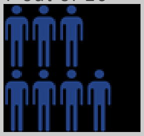
How many out of 10.000 people who take this test have a complication?	None	100 out of 10.000	1 out of 10.000
How often do you need to take the test?	Every 10 years	Every 2 years	Every 5 years
	CBC_Random7=1	CBC_Random7=2	CBC_Random7=3

If you could choose between the test you chose or not to be screened for colorectal cancer, what would you prefer?

CBC_Random7_none=1 I would still prefer the test I chose above

CBC_Random7_none=2 I would prefer not to be screened

0% 100%

CBC_Random8

Imagine that you can choose how you will be screened for colorectal cancer. Please look at the screeningtests below and select the test you prefer by clicking the button below this test.

How do you need to prepare?	Before the test you need to take enemas which cause diarrhoea to empty your colon.	No preparation required.	Before the test you need to take enemas which cause diarrhoea to empty your colon.
How is the test done?	For 2 consecutive days you need to take samples of your stool with applicator sticks that are included in the test package and return them to your doctor. This test is done at home.	A long flexible tube with a small camera is inserted through the anus into the full colon. During the examination you will be sedated. This test is done at a hospital.	A short flexible tube with a small camera is inserted through the anus into the last part of the colon. This test is done at a hospital.
How many out of 10 people with cancer, would the test correctly identify?	8 out of 10 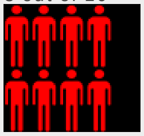	10 out of 10 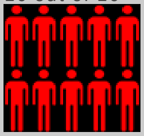	9 out of 10 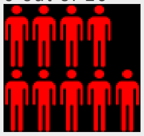
How many out of 10 people without cancer, would the test correctly identify?	7 out of 10 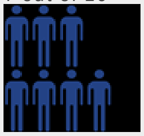	10 out of 10 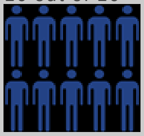	8 out of 10 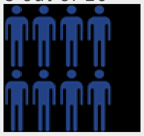
How many out of 10.000 people who take this test have a complication?	10 out of 10.000	None	100 out of 10.000
How often do you need to take the test?	Every 10 years	Every year	Every 5 years
	CBC_Random8=1	CBC_Random8=2	CBC_Random8=3

If you could choose between the test you chose or not to be screened for colorectal cancer, what would you prefer?

CBC_Random8_none=1 I would still prefer the test I chose above

CBC_Random8_none=2 I would prefer not to be screened

0% 100%

CBC_Fixed2

Imagine that you can choose how you will be screened for colorectal cancer. Please look at the screeningtests below and select the test you prefer by clicking the button below this test.

How do you need to prepare?	For 3 days you need to alter your diet and medication. Before the test you need to take laxatives which cause diarrhoea to empty your colon.	Before the test you need to take enemas which cause diarrhoea to empty your colon.	No preparation required.
How is the test done?	A long flexible tube with a small camera is inserted through the anus into the full colon. During the examination you will be sedated. This test is done at a hospital.	A short flexible tube with a small camera is inserted through the anus into the last part of the colon. This test is done at a hospital.	For 2 consecutive days you need to take samples of your stool with applicator sticks that are included in the test package and return them to your doctor. This test is done at home.
How many out of 10 people <u>with</u> cancer, would the test correctly identify?	7 out of 10 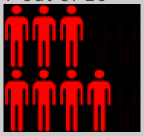	8 out of 10 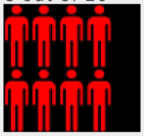	10 out of 10 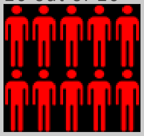
How many out of 10 people <u>without</u> cancer, would the test correctly identify?	8 out of 10 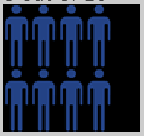	9 out of 10 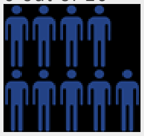	10 out of 10 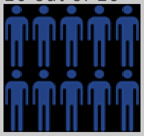
How many out of 10.000 people who take this test have a complication?	100 out of 10.000	10 out of 10.000	1 out of 10.000
How often do you need to take the test?	Every 2 years	Every 5 years	Every 10 years
	CBC_Fixed2=1	CBC_Fixed2=2	CBC_Fixed2=3

If you could choose between the test you chose or not to be screened for colorectal cancer, what would you prefer?

CBC_Fixed2_none=1 I would still prefer the test I chose above

CBC_Fixed2_none=2 I would prefer not to be screened

0% 100%

CBC_Random9

Imagine that you can choose how you will be screened for colorectal cancer. Please look at the screeningtests below and select the test you prefer by clicking the button below this test.

How do you need to prepare?	Before the test you need to take laxatives which cause diarrhoea to empty your colon.	No preparation required.	For 3 days you need to alter your diet and medication. Before the test you need to take laxatives which cause diarrhoea to empty your colon.
How is the test done?	A long flexible tube with a small camera is inserted through the anus into the full colon. During the examination you will be sedated. This test is done at a hospital.	A short flexible tube with a small camera is inserted through the anus into the last part of the colon. This test is done at a hospital.	For 2 consecutive days you need to take samples of your stool with applicator sticks that are included in the test package and return them to your doctor. This test is done at home.
How many out of 10 people <u>with</u> cancer, would the test correctly identify?	8 out of 10 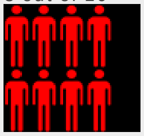	7 out of 10 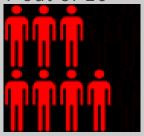	10 out of 10 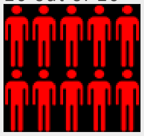
How many out of 10 people <u>without</u> cancer, would the test correctly identify?	9 out of 10 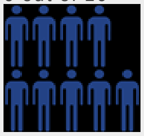	7 out of 10 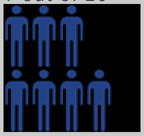	8 out of 10 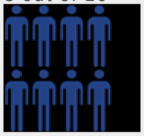
How many out of 10.000 people who take this test have a complication?	100 out of 10.000	1 out of 10.000	10 out of 10.000
How often do you need to take the test?	Every year	Every 2 years	Every 5 years
	CBC_Random9=1	CBC_Random9=2	CBC_Random9=3

If you could choose between the test you chose or not to be screened for colorectal cancer, what would you prefer?

CBC_Random9_none=1 I would still prefer the test I chose above

CBC_Random9_none=2 I would prefer not to be screened

0% 100%

CBC_Random10

Imagine that you can choose how you will be screened for colorectal cancer. Please look at the screeningtests below and select the test you prefer by clicking the button below this test.

How do you need to prepare?	No preparation required.	Before the test you need to take laxatives which cause diarrhoea to empty your colon.	Before the test you need to take laxatives which cause diarrhoea to empty your colon.
How is the test done?	For 2 consecutive days you need to take samples of your stool with applicator sticks that are included in the test package and return them to your doctor. This test is done at home.	You need to swallow a pill that leaves your body through faeces after several hours. Your test results are wirelessly sent to your physician. This test is done at home.	You need to swallow a pill that leaves your body through faeces after several hours. Your test results are wirelessly sent to your physician. This test is done at home.
How many out of 10 people <u>with</u> cancer, would the test correctly identify?	9 out of 10 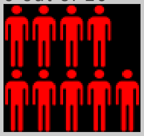	7 out of 10 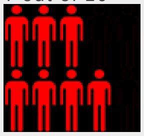	10 out of 10 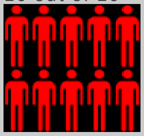
How many out of 10 people <u>without</u> cancer, would the test correctly identify?	9 out of 10 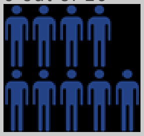	8 out of 10 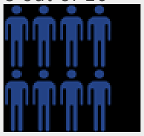	7 out of 10 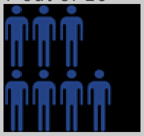
How many out of 10.000 people who take this test have a complication?	None	1 out of 10.000	100 out of 10.000
How often do you need to take the test?	Every 10 years	Every 5 years	Every 2 years
	CBC_Random10=1	CBC_Random10=2	CBC_Random10=3

If you could choose between the test you chose or not to be screened for colorectal cancer, what would you prefer?

CBC_Random10_none=1 I would still prefer the test I chose above

CBC_Random10_none=2 I would prefer not to be screened

0% 100%

CBC_Random11

Imagine that you can choose how you will be screened for colorectal cancer. Please look at the screeningtests below and select the test you prefer by clicking the button below this test.

How do you need to prepare?	Before the test you need to take laxatives which cause diarrhoea to empty your colon.	Before the test you need to take enemas which cause diarrhoea to empty your colon.	For 3 days you need to alter your diet and medication. Before the test you need to take laxatives which cause diarrhoea to empty your colon.
How is the test done?	A short flexible tube with a small camera is inserted through the anus into the last part of the colon. This test is done at a hospital.	A long flexible tube with a small camera is inserted through the anus into the full colon. During the examination you will be sedated. This test is done at a hospital.	You need to swallow a pill that leaves your body through faeces after several hours. Your test results are wirelessly sent to your physician. This test is done at home.
How many out of 10 people with cancer, would the test correctly identify?	7 out of 10 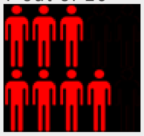	8 out of 10 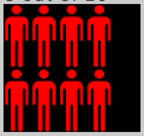	9 out of 10 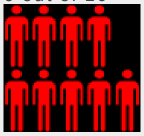
How many out of 10 people without cancer, would the test correctly identify?	7 out of 10 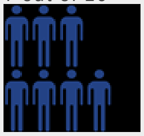	10 out of 10 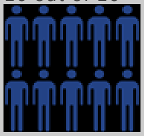	9 out of 10 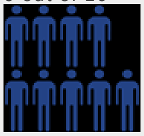
How many out of 10.000 people who take this test have a complication?	None	10 out of 10.000	10 out of 10.000
How often do you need to take the test?	Every 5 years	Every year	Every 10 years
	CBC_Random11=1	CBC_Random11=2	CBC_Random11=3

If you could choose between the test you chose or not to be screened for colorectal cancer, what would you prefer?

CBC_Random11_none=1 I would still prefer the test I chose above

CBC_Random11_none=2 I would prefer not to be screened

0% 100%

CBC_Random12

Imagine that you can choose how you will be screened for colorectal cancer. Please look at the screeningtests below and select the test you prefer by clicking the button below this test.

How do you need to prepare?	For 3 days you need to alter your diet and medication. Before the test you need to take laxatives which cause diarrhoea to empty your colon.	Before the test you need to take enemas which cause diarrhoea to empty your colon.	No preparation required.
How is the test done?	A short flexible tube with a small camera is inserted through the anus into the last part of the colon. This test is done at a hospital.	A long flexible tube with a small camera is inserted through the anus into the full colon. During the examination you will be sedated. This test is done at a hospital.	For 2 consecutive days you need to take samples of your stool with applicator sticks that are included in the test package and return them to your doctor. This test is done at home.
How many out of 10 people <u>with</u> cancer, would the test correctly identify?	8 out of 10 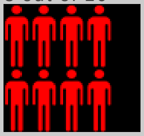	9 out of 10 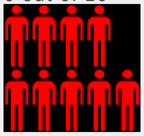	10 out of 10 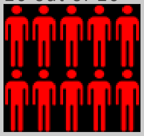
How many out of 10 people <u>without</u> cancer, would the test correctly identify?	9 out of 10 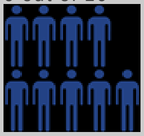	8 out of 10 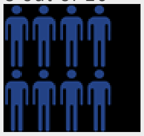	7 out of 10 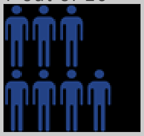
How many out of 10.000 people who take this test have a complication?	100 out of 10.000	1 out of 10.000	None
How often do you need to take the test?	Every 10 years	Every 2 years	Every 10 years
	CBC_Random12=1	CBC_Random12=2	CBC_Random12=3

If you could choose between the test you chose or not to be screened for colorectal cancer, what would you prefer?

CBC_Random12_none=1 I would still prefer the test I chose above

CBC_Random12_none=2 I would prefer not to be screened

0% 100%

CBC_Random13

Imagine that you can choose how you will be screened for colorectal cancer. Please look at the screeningtests below and select the test you prefer by clicking the button below this test.

How do you need to prepare?	No preparation required.	Before the test you need to take laxatives which cause diarrhoea to empty your colon.	Before the test you need to take enemas which cause diarrhoea to empty your colon.
How is the test done?	A short flexible tube with a small camera is inserted through the anus into the last part of the colon. This test is done at a hospital.	You need to swallow a pill that leaves your body through faeces after several hours. Your test results are wirelessly sent to your physician. This test is done at home.	A long flexible tube with a small camera is inserted through the anus into the full colon. During the examination you will be sedated. This test is done at a hospital.
How many out of 10 people with cancer, would the test correctly identify?	10 out of 10 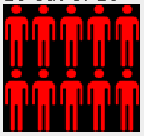	9 out of 10 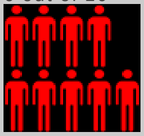	7 out of 10 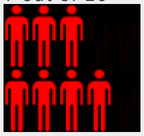
How many out of 10 people without cancer, would the test correctly identify?	9 out of 10 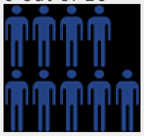	10 out of 10 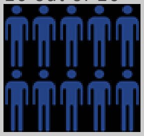	9 out of 10 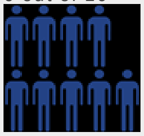
How many out of 10.000 people who take this test have a complication?	1 out of 10.000	100 out of 10.000	None
How often do you need to take the test?	Every 5 years	Every year	Every 2 years
	CBC_Random13=1	CBC_Random13=2	CBC_Random13=3

If you could choose between the test you chose or not to be screened for colorectal cancer, what would you prefer?

CBC_Random13_none=1 I would still prefer the test I chose above

CBC_Random13_none=2 I would prefer not to be screened

0% 100%

CBC_Random14

Imagine that you can choose how you will be screened for colorectal cancer. Please look at the screeningtests below and select the test you prefer by clicking the button below this test.

How do you need to prepare?	For 3 days you need to alter your diet and medication. Before the test you need to take laxatives which cause diarrhoea to empty your colon.	No preparation required.	Before the test you need to take enemas which cause diarrhoea to empty your colon.
How is the test done?	A long flexible tube with a small camera is inserted through the anus into the full colon. During the examination you will be sedated. This test is done at a hospital.	For 2 consecutive days you need to take samples of your stool with applicator sticks that are included in the test package and return them to your doctor. This test is done at home.	A short flexible tube with a small camera is inserted through the anus into the last part of the colon. This test is done at a hospital.
How many out of 10 people <u>with</u> cancer, would the test correctly identify?	9 out of 10 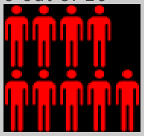	8 out of 10 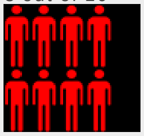	10 out of 10 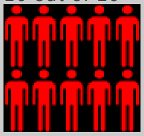
How many out of 10 people <u>without</u> cancer, would the test correctly identify?	10 out of 10 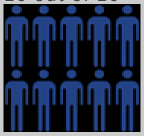	8 out of 10 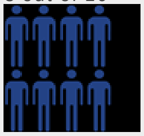	7 out of 10 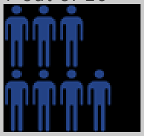
How many out of 10.000 people who take this test have a complication?	None	10 out of 10.000	1 out of 10.000
How often do you need to take the test?	Every 5 years	Every year	Every year
	CBC_Random14=1	CBC_Random14=2	CBC_Random14=3

If you could choose between the test you chose or not to be screened for colorectal cancer, what would you prefer?

CBC_Random14_none=1 I would still prefer the test I chose above

CBC_Random14_none=2 I would prefer not to be screened

0% 100%

Gender

Question 17.

What is your gender?

Gender=1 Male

Gender=2 Female

0% 100%

Age

Question 18.

What is your age?

0% 100%

Status

Question 19.

What is your marital status?

Status=1 Married

Status=2 Not married

0% 100%

Employment

Question 20.

What is your current employment status?

Employment=1
- Employed full-time

Employment=2
- Employed part-time

Employment=3
- Self-employed

Employment=4
- Homemaker

Employment=5
- Unemployed

Employment=6
- Retired

0% 100%

Education

Question 21.

What is the highest level of education you completed?

Education=1 Public or primary school

Education=2 High school

Education=3 Trade or technical qualification

Education=4 College or university

0% 100%

Family

Question 22.

Do you have a family history (parents, brothers, sisters or children) of colorectal cancer?

Family=1
Yes

Family=2
No

Family=3
Do not know

0% 100%

Experience

Question 23.

Do you have experience with colorectal cancer screening?

Experience=1 Yes

Experience=2 No

0% 100%

Health

Question 24.

How would you say your current health status is?

Health=1 Excellent

Health=2 Very good

Health=3 Good

Health=4 Fair

Health=5 Poor

0% 100%

Perceivedrisk

Question 25.

Do you think you are at risk of developing colorectal cancer?

Perceivedrisk=1 Yes

Perceivedrisk=2 No

0% 100%

Finish

Thank you for your participation!

Your data is saved. If you have any questions about this questionnaire, please contact:

J.M. Fermont
j.m.fermont@student.utwente.nl

School for Management and Governance
Dept. Health Technology and Services Research
http://www.utwente.nl/education/mb

Click on the following link to close this window and visit Survey Sampling International.

Powered by Sawtooth Software, Inc.

0% 100%

Finish2

0% 100%
